# The Tumor Suppressor Kinase LKB1: Metabolic Nexus

**DOI:** 10.3389/fcell.2022.881297

**Published:** 2022-04-28

**Authors:** Mohammed Bourouh, Paola A. Marignani

**Affiliations:** Department of Biochemistry and Molecular Biology, Faculty of Medicine, Dalhousie University Halifax, Halifax, NS, Canada

**Keywords:** LKB1, AMPK, mTOR, tumor suppressor, cancer metabolism, glycolysis, lung cancer

## Abstract

Liver kinase B1 (LKB1) is a multitasking tumor suppressor kinase that is implicated in multiple malignancies such as lung, gastrointestinal, pancreatic, and breast. *LKB1* was first identified as the gene responsible for Peutz-Jeghers syndrome (PJS) characterized by hamartomatous polyps and oral mucotaneous pigmentation. LKB1 functions to activate AMP-activated protein kinase (AMPK) during energy stress to shift metabolic processes from active anabolic pathways to active catabolic pathways to generate ATP. Genetic loss or inactivation of *LKB1* promotes metabolic reprogramming and metabolic adaptations of cancer cells that fuel increased growth and division rates. As a result, *LKB1* loss is associated with increased aggressiveness and treatment options for patients with *LKB1* mutant tumors are limited. Recently, there has been new insights into the role LKB1 has on metabolic regulation and the identification of potential vulnerabilities in *LKB1* mutant tumors. In this review, we discuss the tumor suppressive role of *LKB1* and the impact *LKB1* loss has on metabolic reprograming in cancer cells, with a focus on lung cancer. We also discuss potential therapeutic avenues to treat malignancies associated with *LKB1* loss by targeting aberrant metabolic pathways associated with *LKB1* loss.

## Introduction

Metabolism is the outcome of key processes and reactions that generate energy to maintain cellular life. Metabolic processes serve to produce adenosine triphosphate (ATP) to meet the energetic demands of a cell, and the intermediates from these processes are used to generate biomolecules (Hanahan and Weinberg, 2011; Pavlova and Thompson, 2016). Metabolic reactions can either be anabolic (to buildup) or catabolic (to breakdown) and both these processes must be balanced to maintain the energy supply of cells while preserving biomolecules to sustain cellular function.

A common characteristic of cancer cells is an insatiable demand for energy in order to meet the needs for growth and proliferation. Cancer cells will take control over multiple signaling networks to reprogram metabolic pathways that enable cancer cells to synthesize biomolecules and adapt to survive under elevated reactive oxygen species (ROS) (Hanahan and Weinberg, 2011; Pavlova and Thompson, 2016). The mechanism behind metabolic reprogramming involves genetic adaptations through mutation of tumor suppressor genes and oncogenes that allow metabolic processes to be deregulated, leading to increased proliferation rate and survival of cancer cells. One such gene is the tumor suppressor serine/threonine kinase liver kinase B1 (*LKB1*), also known as serine-threonine kinase 11 (*STK11*).

*LKB1* is implicated in multiple malignancies where it is often lost or inactivated. *LKB1* was first identified as the gene responsible for Peutz-Jeghers syndrome (PJS), a dominant disorder characterized by benign hamartomatous polyps in the gastrointestinal tract and mucocutaneous melanin pigmentation (Peutz, 1921; Jeghers et al., 1949; Hemminki et al., 1997). Germline mutations in *LKB1* that lead to the development of PJS result in loss of function of *LKB1* through truncations, deletions, or direct mutations to the kinase domain abolishing LKB1 kinase activity (Mehenni et al., 1998; Tiainen et al., 1999; Ylikorkala et al., 1999; Boudeau et al., 2003a). PJS patients have an increased risk of developing different malignancies, primarily in the gastrointestinal tract; colon, gastric, and intestinal cancers (Giardiello et al., 2000; Karuman et al., 2001), and are also susceptible to malignancies of the breast, lung, uterus, ovaries, cervix, and testes (Avizienyte et al., 1998; Nishioka et al., 1999; Boardman et al., 2000; Sanchez-Cespedes et al., 2002; Shen et al., 2002).

While *LKB1* mutations in PJS are associated with an increased risk of developing cancer, *LKB1* somatic mutations leading to malignancies are rare. It is surprising then, that the exception is in non-small cell lung cancer (NSCLC), where *LKB1* loss is implicated in 30% of cases (Sanchez-Cespedes, 2007; Ding et al., 2008; Gill et al., 2011). Furthermore, *LKB1* haploinsufficiency has been observed in the pancreas (Morton et al., 2010), breast (Shen et al., 2002), endometrial (Contreras et al., 2008) and liver adenocarcinoma (Kim and Chen, 2004) although very infrequent.

The tumor suppressor function of LKB1 has largely been attributed to the phosphorylation and activation of the energy sensor AMP-activated protein kinase (AMPK) in response to nutrient availability and energy stress. Here, the LKB1-AMPK axis shifts cellular metabolism from active anabolic pathways to active catabolic pathways to correct the energy imbalance (Hardie, 2005).

When LKB1 activity is abolished, the mechanism regulating metabolic pathways is eliminated. Loss of *LKB1* leads to increased glucose uptake and increased activity of aerobic glycolysis, commonly known as the Warburg effect (Warburg et al., 1927). Furthermore, loss of *LKB1* also leads to increased ROS that needs to be quenched to prevent damage to macromolecules. The survival of cancer cells relies on meeting the energy demand and adapting to the increased ROS produced. In this review, we discuss the tumor suppressive role of *LKB1* as a metabolic nexus, and how it is implicated in metabolic regulation, focusing on lung cancer. We also discuss the impact loss of *LKB1* has on metabolic reprograming and tumor progression and potential therapeutic avenues to treat *LKB1* deficient cancers by targeting aberrant metabolic pathways.

## *LKB1* Mutations in Lung Cancer

*LKB1* is spontaneously mutated most frequently in lung cancer patients is associated with increased aggressiveness (Calles et al., 2015; Lin et al., 2021). *LKB1* loss or inactivation is observed in 30% of lung adenocarcinoma. Lung adenocarcinoma (LUAD) is the most common type of lung cancer, accounting for 45% of cases. LUAD cases are stratified based on oncogenic mutation with ∼60% of LUAD cases associated with *KRAS* and *EGFR* mutations (Sun et al., 2007; Collisson et al., 2014). *EGFR* mutations are more common in never smokers with the most frequently observed mutations being exon 19 deletions, and the point mutation codon 858 (L858R). *KRAS* oncogenic mutations are present in smokers and are often the result of base substitutions at codons 12 (91%), 13 (6%), and 61 (2%). *KRAS* codon 12 mutations result in amino acid substitutions of glutamine to either cysteine (G12C, 44%), valine (G12V, 23%), or aspartic acid (G12D, 17%) being the most common (Ostrem et al., 2013; Cox et al., 2014). KRAS is a small GTPase that promotes activation of the MAPK pathway to promote cell growth and survival (Burotto et al., 2014). Lung adenocarcinoma patients that present with *KRAS* mutations have a higher mutational burden and co-occurring mutations with tumor suppressors genes. Different co-occurring mutations with *KRAS* exhibit unique tumor behaviors and have different gene expression profiles (Skoulidis et al., 2015).

The most studied *Kras* lung cancer mouse model is the *LSL-Kras* ^*G12D*^ mouse model. Using a transcriptional STOP element flanked by *loxP* sites, expression of *Kras* ^*G12D*^ can be induced in multiple ways: tissue and cell-specific promoters driving Cre expression, inhalation of adeno-Cre (Ad5-CMV-Cre), or intratracheal administration of Ad5-CMV-Cre. With Ad5-CMV-Cre administration, lung cancer development can be followed in a time-dependent manner. Furthermore, the clonality of cancer development can also be studied. Expression of *Kras* in the lung caused characteristics of early adenocarcinoma, where lungs presented with atypical adenomatous hyperplasia (AAH), epithelial hyperplasia (EH), and adenomas. Although this mouse model recapitulated early disease histopathologies of lung cancer, it did not recapitulate late stages. When loss of function mutations of *Tp53* were combined with *Kras* oncogenic mutations, late-stage disease progression of lung adenocarcinoma was observed; nuclear atypia, elicit stromal desmoplasia, invasion, and metastasis (Jackson et al., 2005). It was not until *Lkb1* was co-mutated with *Kras* that lung adenocarcinoma disease progression exhibited a similar pattern and severity to human disease.

Mutations in other tumor suppressors (*RB1*, *CDKN2A*, *SMARCA4/BRG1*) are also frequently implicated in lung cancer. *TP53* is the most frequently mutated tumor suppressor in many cancers including lung cancer where it is mutated in ∼70% of cases (Sanchez-Cespedes, 2007). *KRAS* mutant lung adenocarcinoma is often associated with a mutation in *CDKN2A* or *LKB1*. Mouse models of lung cancer typically use three different genotypes to recapitulate LUAD; *Kras;Tp53* (KP), *Kras;Cdkn2a* (KC), and *Kras;Lkb1* (KL). Each model exhibits different characteristics, severity, and aggressiveness of lung adenocarcinomas with different microenvironments, gene expression signatures, and responses to therapies (Skoulidis et al., 2015).

Early work on the KL mouse model characterized LUAD development compared to the KP genotype. The KL genotype is the only genetic combination to fully recapitulate human lung adenocarcinoma in mice, showing all histological subgroups: squamous cell carcinoma, large cell carcinoma, adenocarcinoma, and adenosquamous carcinoma (Ji et al., 2007; Chong and Jänne, 2013; Shaw and Engelman, 2013). Adenosquamous and squamous subtypes are not seen in KP or KC models. *Lkb1* ectopic expression in KL, KC, or KP tumors significantly reduced growth and induced apoptosis, further demonstrating the functional classification of *Lkb1* as a tumor suppressor gene (Ji et al., 2007).

## The LKB1 Kinase

*LKB1* is a conserved, ubiquitously expressed multitasking serine/threonine kinase with tumor suppressor function (Marignani et al., 2010). *LKB1* is a member of the Ca^2+^-calmodulin dependent protein kinase family (Marignani, 2005) with orthologues in frogs, mice, worms, and flies (Smith et al., 1999; Watts et al., 2000; Martin and St Johnston, 2003). The human *LKB1* gene maps to chromosome 19p13.3 and is 23 kb long, consisting of 10 exons of which exons 1–9 are coding and exon 10 is non-coding. *LKB1* is transcribed in the telomere to centromere direction and generates a 50 kDa protein. *LKB1* is ubiquitously expressed in mice and humans with tissue-specific differences in overall abundance (Towler et al., 2008). In mice, Lkb1 protein is most abundant in embryonic and extra-embryonic tissues. Later in development, Lkb1 protein is concentrated in heart, esophagus, pancreas, kidney, colon, lung, small intestines, and stomach tissues (Luukko et al., 1999; Rowan et al., 1999). In adult mice, Lkb1 protein levels are most abundant in epithelial tissues, follicles, and corpus luteum of the ovary, seminiferous tubules of the testis, skeletal muscle monocytes, and glial cells (Rowan et al., 1999; Conde et al., 2007).

**FIGURE 1:**
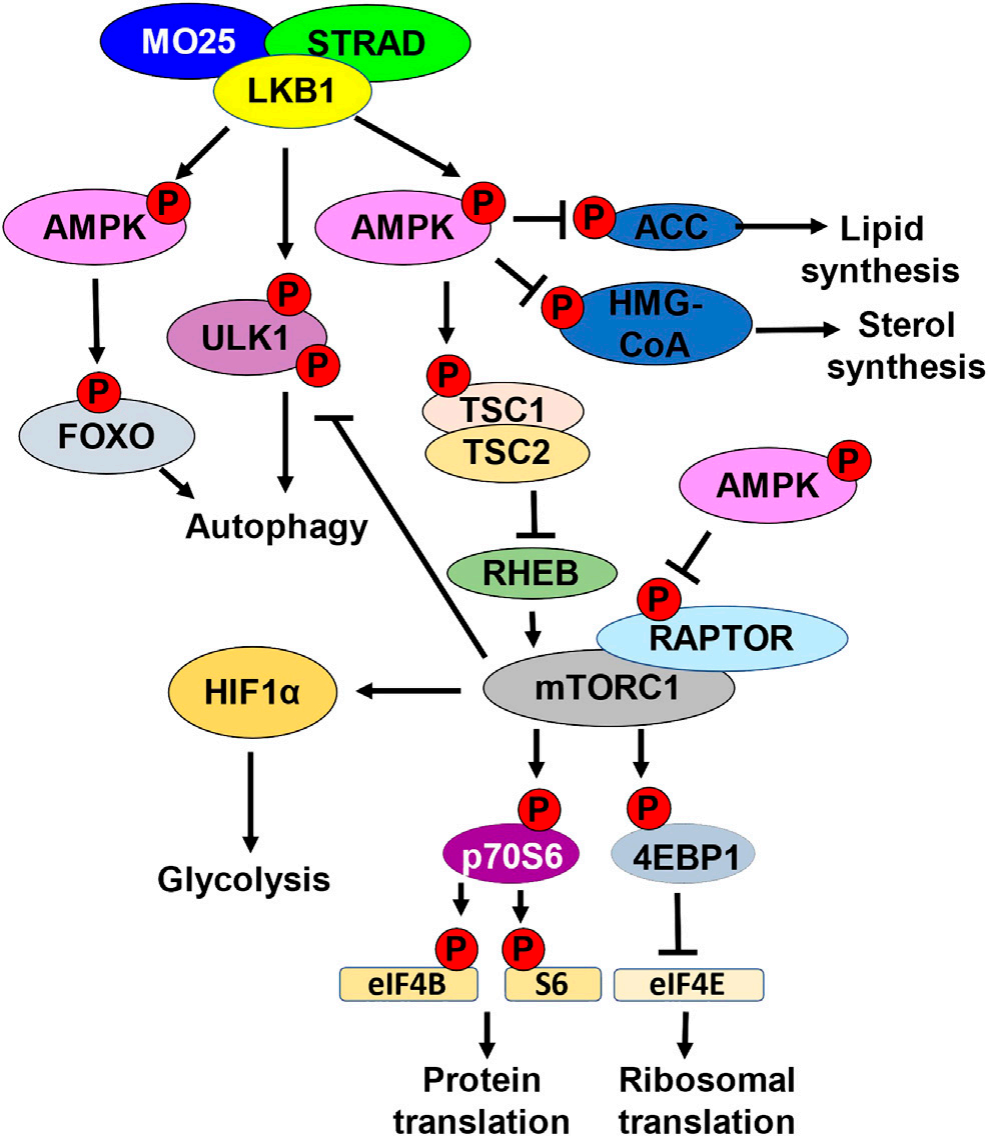
LKB1 metabolic nexus. Schematic representation of downstream LKB1 signaling. LKB1 in complex with STRAD and MO25 phosphorylates and activates AMPK. AMPK phosphorylates and inhibits ACC, inhibiting lipid synthesis. AMPK phosphorylates and inhibits HMG-CoA, inhibiting sterol synthesis. Active AMPK also regulates autophagy by phosphorylating and activating FOXO. LKB1 can directly promote autophagy by phosphorylating ULK1. LKB1 activation of AMPK also inhibits mTORC1 kinase activation. mTORC1 kinase phosphorylates and activates p70S6. p70S6 phosphorylates and activates eIF4B and S6 kinase, promoting protein translation. mTORC1 also phosphorylates and inhibits 4EBP1, the inhibitor of eIF4E. eIF4e activation leads to increased ribosomal translation. mTORC1 can also inhibits autophagy by phosphorylating ULK1. mTORC1 promotes glycolysis by upregulating HIF1α. AMPK directly inhibits mTORC1 by phosphorylating RAPTOR. AMPK can also indirectly inhibit mTORC1 by phosphorylating and activating TSC1/TSC2. Active TSC1/2 complex inhibits RHEB.

LKB1 is primarily found in a complex with the pseudokinase STE20-related adaptor (STRAD) (Baas et al., 2003) and the scaffolding protein Mouse protein 25 (MO25) (Boudeau et al., 2003b; Marignani et al., 2007). Unlike other kinases, LKB1 does not become catalytically active through T-loop threonine phosphorylation of the kinase domain, but instead when bound to adaptor proteins STRAD and MO25 (Baas et al., 2003; Boudeau et al., 2003b; Boudeau et al., 2006; Marignani et al., 2007; Dorfman and Macara, 2008). STRAD is a homolog of the STE20 family of kinases but lacks multiple critical residues required for a functional kinase domain (Dorfman and Macara, 2008). Although STRAD lacks a functional kinase domain, STRAD adopts an active conformation when bound to ATP (Zeqiraj et al., 2009). The binding of STRAD to MO25 enhances the affinity of STRAD to ATP. Furthermore, STRAD binding to ATP and MO25 are required for LKB1 catalytic activation (Baas et al., 2003; Boudeau et al., 2003b; Boudeau et al., 2006; Dorfman and Macara, 2008). LKB1 when in complex with STRAD and MO25 increases LKB1 catalytic activity by approximately 10 fold compared to LKB1 alone (Boudeau et al., 2003b) (Figure 1).

In addition to regulating the activity of LKB1, STRAD also regulates LKB1 subcellular localization, particularly nuclear-cytoplasmic localization. Individually, STRAD and MO25 can freely diffuse through nuclear pores and thus are localized both in the nucleus and cytoplasm. When *STRAD* and *MO25* are co-expressed, they exhibit exclusively cytoplasmic localization. LKB1 contains an N-terminal nuclear localization signal (NLS) directing LKB1 to the nucleus *via* an importin α/β mechanism. STRAD competes with importin α/β for binding to LKB1, and therefore binding of STRAD to LKB1 sequesters LKB1 in the cytoplasm. Co-expression of *LKB1* with *STRAD* and *MO25* causes LKB1 to localize in the cytoplasm (Baas et al., 2003; Boudeau et al., 2003b). STRAD promotes LKB1 nuclear export in a CRM1 and exportin7 dependent manner (Dorfman and Macara, 2008).

Oncogenic mutations generally occur in the kinase domain of *LKB1* and abolish kinase activity. *LKB1* introduction to cancer cell lines that do not express *LKB1* results in a G1 arrest (Tiainen et al., 1999; Tiainen et al., 2002). Expression of catalytic deficient *LKB1* mutants, where the mutations are found within the kinase domain, does not result in a G1 arrest, suggesting that catalytic activity is required for tumor suppressor function (Scott et al., 2007). Furthermore, *LKB1* mutations that abolish the binding to STRAD-MO25 also do not exhibit cell cycle arrest functions suggesting that the binding of STRAD-MO25 to LKB1 is required for the cell cycle arrest function of LKB1 (Tiainen et al., 1999; Tiainen et al., 2002; Scott et al., 2007). Interestingly, the expression of *LKB1* with a catalytically deficient point mutation, *LKB1* ^*D194A*^, resulted in the expression of genes important in cell cycle progression (*CYCD1*, *RB1*, *CYCE*, and *CYCA*). This suggests that catalytic deficient mutants of LKB1 can promote cell growth through kinase-independent functions (Scott et al., 2007).

## Characterization of *LKB1* Using Mouse Models

The early developmental link between *LKB1* and PJS motivated the generation of *Lkb1* loss of function alleles in mice to study the function of *Lkb1* in disease. Homozygous loss of *Lkb1* in mice resulted in embryonic lethality at midgestation. This lethality was attributed to abnormal *Vegf* regulation, where *Vegf* was expressed at higher levels compared to wild-type mice. *Vegf* expression is regulated in part through hypoxia-induced factor (Hif1α), suggesting that loss of *Lkb1* results in metabolic stress (Ylikorkala et al., 2001).

To better characterize Lkb1 function *in vivo*, conditional knockout alleles were generated to study the consequence of *Lkb1* loss in tissue specific manner. Conditional *Lkb1* knockout (KO) alleles were generated to induce *Lkb1* KO using Cre-recombinase (Bardeesy et al., 2002). Characterization of these alleles confirmed earlier observations that *Lkb1* loss is embryonic lethal and that *Lkb1* heterozygotes developed hamartomatous polyps like PJS patients (Bardeesy et al., 2002). Furthermore, DePinho’s group determined that *Lkb1* heterozygous mice were more susceptible to carcinogenesis when exposed to 7,12-dimethylbenz(a)anthracene (DMBA) (Bardeesy et al., 2002).

This mouse model was used to identify the role of Lkb1 in energy metabolism. The Alessi group studied the effects of *Lkb1* KO in muscle tissues by inducing *Lkb1* KO using Cre driven under the *muscle creatine kinase (MCK)* promoter. Expression of *MCK-Cre* excised *Lkb1* in heart and skeletal muscle. They found that the AMP:ATP ratios were significantly elevated compared to control mice. This suggested that Lkb1 was involved in correcting the metabolic imbalance and that when Lkb1 activity is lost, muscle cells were not able to generate ATP to correct the imbalance (Sakamoto et al., 2005). This work led to the connection between LKB1 and energy metabolism. Later, it was discovered that LKB1 promotes the phosphorylation and activation of the AMPK family of kinases (Hawley et al., 2003; Lizcano et al., 2004).

## LKB1 Activates the AMPK Family of Kinases

LKB1 functions upstream of the AMPK of kinases family, which consists of AMPK, and 12 other related kinases termed the AMPK-related kinases (ARKs); novel (Nu) AMP related kinase 1 and 2 (NUAK1,2), salt inducible kinase 1–3 (SIK1-3), microtubule affinity regulating kinases 1–4 (MARK1-4), brain selective kinases 1 and 2 (BRSK1,2), and maternal embryonic leucine zipper kinase (MELK) (Scott et al., 2007).

Before the association of LKB1 with energy metabolism, the *C.elegans* ortholog of *LKB1* (*Par-4)* was implicated in cell polarity. Par-4 is asymmetrically localized during the first embryonic division, providing key signals for fate decisions in subsequent cell divisions (Watts et al., 2000). A role for LKB1 in regulating cell polarization was later observed in mammalian cells, suggesting evolutionary conserved function. Furthermore, the discovery that LKB1 phosphorylates and activates the ARK kinases in addition to AMPK provided insights into the potential mechanism behind the cell polarity related function of LKB1 (Lizcano et al., 2004). Both the MARK and BRSK kinases regulate microtubule dynamics and contribute to regulate cell polarity through LKB1 phosphorylation and activation (Kojima et al., 2007; Nakano and Takashima, 2012). MARK proteins phosphorylate and inhibit microtubule associated proteins (MAPs) causing microtubule depolymerization and reorganization (Kojima et al., 2007). A genetic screen in HEK293T cells identified the Hippo pathway protein YAP as a mediator of LKB1-MARK axis, implicating LKB1 in regulate organ size (Mohseni et al., 2014).

The tumor suppressor function of LKB1 is also partially mediated to the ARK kinases. LKB1 phosphorylation and activation of NUAK1 promotes cell cycle arrest in response to UV induced DNA damage. LKB1 and NUAK1 can phosphorylate cyclin-dependent kinase inhibitor 1A (CDKN1A), which inhibits cyclin-CDK complexes preventing G1/S transition and leads to a cell cycle arrest in a TP53 dependent mechanism (Zeng and Berger, 2006; Esteve-Puig et al., 2014). NUAK 1 and 2 are also implicated in glucose tolerance and attenuation of insulin signaling in muscle cells and regulate cell motility and muscle contraction through activation of myosin (Koh et al., 2010; Zagórska et al., 2010; Vallenius et al., 2011).

Finally, the LKB1-SIK axis plays a role in regulating metabolism. SIK phosphorylation of cAMP response element binding protein (CREB) and CREB regulated transcription co-activator (CRTC) regulates multiple biological processes including metabolism, cell differentiation, and proliferation (Gao et al., 2018). LKB1 activation of SIK kinases inhibits gluconeogenesis in liver cells, and the LKB1-SIK axis promotes growth and differentiation of adipose tissue (Patel et al., 2014; Darling and Cohen, 2021). Furthermore, LKB1-SIK axis can promote GLUT4 mediated glucose import in muscle and adipose tissues by phosphorylating and inhibiting CRTC2/3 (Stringer et al., 2015). SIK1 and SIK3 inhibit the expression of lipogenic genes, thereby reducing lipogenesis (Du et al., 2008; Sun et al., 2020).

The different and numerous ARK kinases highlight the diverse and cell specific functions of LKB1. LKB1 can directly phosphorylate and activate all members of the AMPK family except MELK. In this way, LKB1 acts as a master regulatory kinase acting through the AMPK family of kinases in a cell specific manner to regulate multiple pathways related to metabolism, cell polarity, migration, division, and transcription (Ylikorkala et al., 2001).

## LKB1-AMPK in Metabolic Regulation

The best characterized, and primary target of LKB1 to regulate energy metabolism is AMPK, which is the focus of this review. LKB1 functions upstream of AMPK, the central regulator in maintaining intracellular ATP levels. Upon activation under energy stress, when the AMP:ATP ratio is high, AMPK acts as a metabolic switch to inhibit anabolic (fatty acid and protein synthesis) pathways and promotes the activation of catabolic pathways (glycolysis, fatty acid oxidation, and autophagy) to correct the energy imbalance (Ciccarese et al., 2019). AMPK is a heterotrimeric protein kinase composed of a catalytic *a* subunit, and two regulatory subunits *ß* and *γ*. AMP binds the *γ* subunit promoting a conformational change to remove allosteric inhibition of AMPK and promote its activation with additional Thr172 phosphorylation by upstream kinases. The primary kinase responsible for AMPK activation was identified to be LKB1, providing the first insights that the tumor suppressor function of LKB1 was mediated through AMPK to regulate energy metabolism (Hawley et al., 2003; Woods et al., 2003; Hardie and Alessi, 2013).

One of the direct readouts to assess AMPK activity is phosphorylation of acetyl-CoA carboxylase (ACC) which is the first enzyme in *de novo* lipid synthesis. AMPK-mediated phosphorylation of ACC is an inhibitory post-translational modification, thereby inhibiting fatty acid synthesis (Carling et al., 1987). AMPK also phosphorylates HMG-CoA reductase which plays a role in sterol synthesis (Sato et al., 1993). Fatty acid synthesis is important for cancer cell progression and *LKB1* mutant tumors exhibit elevated gene expression signature of genes involved in lipid synthesis (Carling et al., 1987; Bhatt et al., 2019) (Figure 1).

The LKB1-AMPK axis also inhibits the central integrator of energy and mitogenic signals to cell growth and division, the mammalian target of rapamycin complex 1 (mTORC1) (Corradetti et al., 2004). mTORC1 with its adaptor regulatory-associated protein of mTOR (RAPTOR), is a kinase responsible for promoting the phosphorylation of eukaryotic initiation factor 4E (EIF4E) binding protein (4EBP1), an inhibitor of EIF4E elongation factor, and p70 ribosomal S6 kinase 1 (p70S6). mTORC1 promotes the translation of mRNAs required for cell growth and division, including ribosomal proteins and proteins involved in translation (Thoreen et al., 2012) (Figure 1).

AMPK can indirectly inhibit mTORC1 by phosphorylating and activating tuberous sclerosis complex 2 (TSC2) (Corradetti et al., 2004). TSC2 functions as a heterodimer with TSC1, and together functions to indirectly inhibit mTOR through inhibition of the GTPase RAS homolog enriched in the brain (RHEB). When RHEB is active, RHEB binds and activates mTORC1 using its GTPase activity to induce a conformational change to the mTORC1 complex (Tee et al., 2003). TSC1/TSC2 bind and inhibit RHEB, thereby inhibiting mTORC1. AMPK can also directly inhibit mTORC1 by directly phosphorylating RAPTOR (Jewell et al., 2019). AMPK phosphorylation of RAPTOR promotes 14–3–3 protein binding, inhibiting mTORC1 from interacting with downstream targets 4EBP1 and p70S6 (Nojima et al., 2003) (Figure 1).

A consequence of *LKB1* loss is enhanced mTORC1 activation. mTORC1 promotes cell growth and metabolic changes such as increasing glycolysis and inhibiting autophagy. LKB1-AMPK directly, and indirectly through mTORC1, regulate autophagy. LKB1 directly phosphorylates ULK1 to promote autophagy and plays a role in mitochondrial biogenesis. mTORC1 phosphorylates and inhibits ULK1 inhibiting autophagy, and this is dysregulated in *LKB1* mutant cells. Furthermore, AMPK can promote the transcription of genes involved in autophagy through activation of the transcription factor FOXO (Kim et al., 2011) (Figure 1). Through the regulation of mTORC1, LKB1 and AMPK provide an important regulatory link between cell metabolism and cell growth and division.

## *LKB1* Loss Promotes Metabolic Changes

The LKB1-AMPK-mTORC1 axis is often deregulated in cancer cells, resulting in metabolic changes to support cancer cell growth and division. Cancer cells are often in a state of energy imbalance and have elevated energy requirements to manage high growth and division rates. Although producing less ATP, cancer cells show increased glycolysis rates even in the presence of oxygen. This shift to aerobic glycolysis demonstrates the Warburg effect (Pavlova and Thompson, 2016). LKB1 plays an important role in regulating glycolysis. Cells mutant for *LKB1* exhibit increased glucose uptake and increased extracellular acidification rate (ECAR). ECAR is determined by measuring lactate levels, which is produced by lactate dehydrogenase (LDH) to regenerate NAD+ for glycolysis. Lactate is then exported out of the cell causing acidification of the extracellular space. In the lung cancer cell line A549, which is deficient for *LKB1*, transient expression of *LKB1* resulted in a reduction of ECAR by 20% (Faubert et al., 2014). This suggests that LKB1 is an important regulator of glycolysis and that *LKB1* loss impacts glycolysis rate in cancer cells.

The metabolic reprogramming that occurs under *LKB1* loss is also observed in a Her2 positive *Lkb1* mutant breast cancer mouse model, *Stk11* ^*−/−*^ */NIC*. Loss of *Lkb1* reduces the latency of Her2 positive breast cancer from 197 to 147 days. Loss of *Lkb1* is associated with elevated mTORC1 activation and reduced AMPK activity (Andrade-Vieira et al., 2013). LKB1 acts as a metabolic nexus, connecting AMPK and mTORC1 signaling with downstream metabolic pathways. One such link is how *LKB1* loss impacts glycolysis. AMPK and mTORC1 are important regulators of glycolysis and since *Lkb1* acts upstream of AMPK and mTOR, the Marignani group examined the metabolic changes that occur when *LKB1* is lost. *Stk11* ^*−/−*^ */NIC* tumors exhibit increased branch chain amino acids (BCAA) valine and isoleucine and elevated glutamine and methionine levels and showed lower levels of glutathione, a ROS scavenger suggesting increased ROS stress which promotes tumor development. Furthermore, LDH and pyruvate dehydrogenase (PDH) levels are elevated, indicating elevated glycolysis (Andrade-Vieira et al., 2013).

**FIGURE 2:**
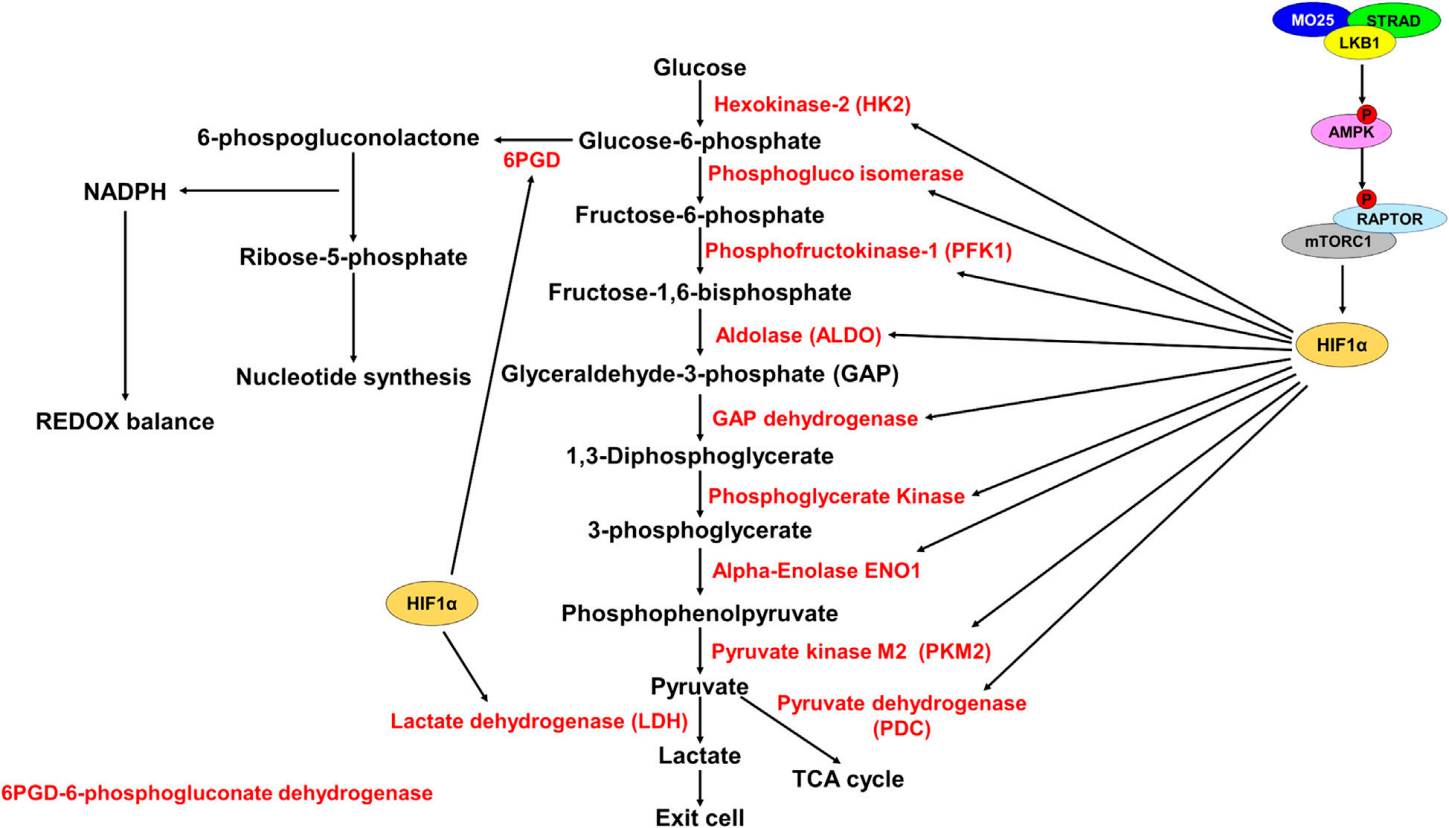
HIF1α promotes expression of glycolytic enzymes. Schematic representation of glycolysis and the pentose phosphate pathway (PPP). LKB1 phosphorylates and activates AMPK which then phosphorylates and inhibits RAPTOR, thereby inhibiting mTORC1. mTORC1 promotes HIF1α upregulation. Active HIF1α promotes expression of glycolytic enzymes (red) increasing glycolysis rate. HIF1α also promotes expression of 6PGD to shunt glucose-6-phosphate to the PPP to generate NADPH for REDOX balance and for synthesis of ribose-5-phosphate for nucleotide synthesis.

In this model of breast cancer, loss of *Lkb1* synergizes with activated Her2, which promotes downstream activation of PI3K and AKT, leading to elevated mTORC1 activity. Elevated mTORC1 promotes metabolic reprogramming, increasing glycolysis by promoting the translation of glucose transporters, and glycolytic enzymes. The resulting increase in glycolysis is observed through elevated lactate (Andrade-Vieira et al., 2013) (Figure 2).

## Glycolysis

Glycolysis is an important metabolic pathway that supports energy and biomolecule production, synthesis of ROS scavengers, and synthesis of nucleotides. Under normal physiological conditions, glucose is oxidized to pyruvate and shunted to the electron transport chain (ETC) for the generation of ATP by oxidative phosphorylation (OXPHOS). Under aerobic conditions, 1 molecule of glucose generates ∼36 ATP and under anaerobic conditions, generates 2 molecules of ATP (Spinelli and Haigis, 2018). Glycolysis generates significantly fewer ATP molecules than OXPHOS but occurs at a much faster rate, therefore increased glycolysis can supply ATP production quicker under anaerobic conditions (Rogatzki et al., 2015). To do this, regeneration of NAD+ is required to support glycolysis. LDH generates NAD+ and lactate from pyruvate and NADH, thus supporting the increased glycolysis and ATP generation requirement (Figure 2).

**FIGURE 3:**
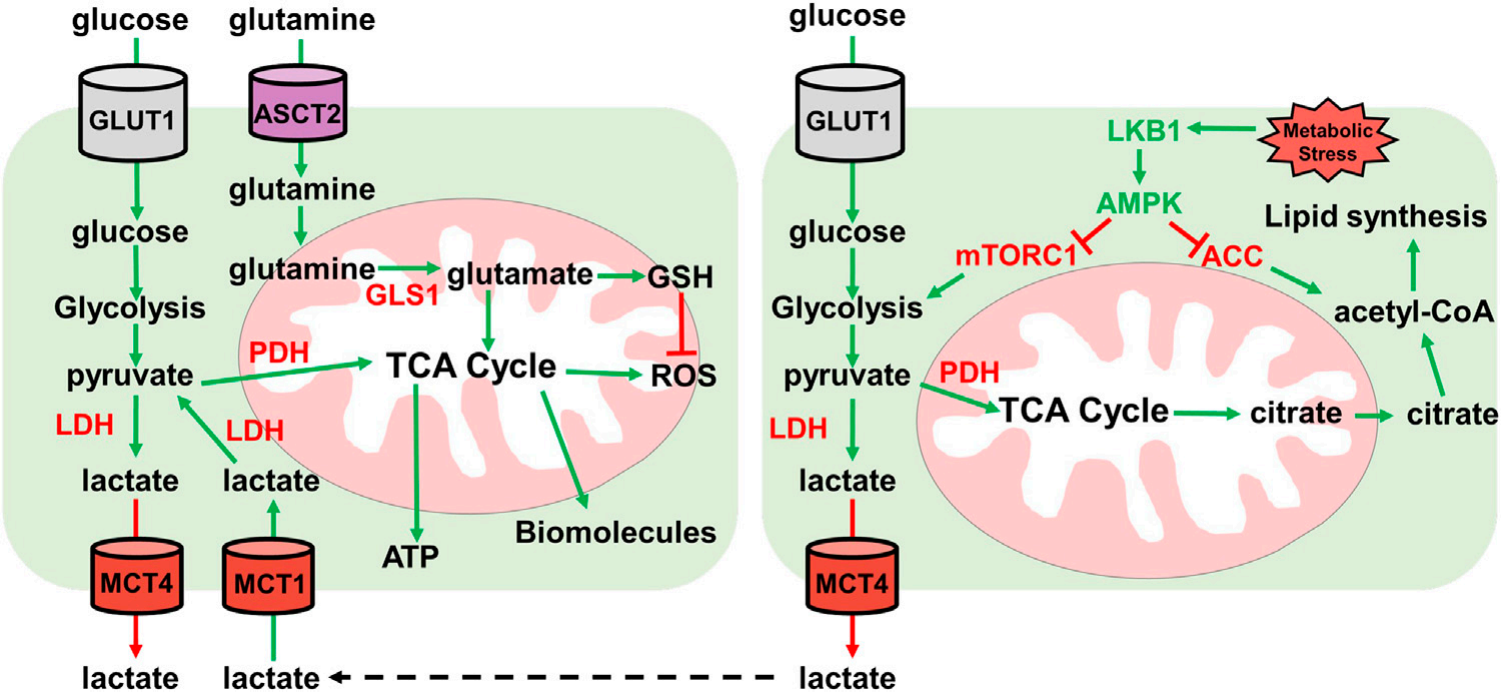
Lactate and glutamine metabolism. Overview of lactate and glutamine metabolism. Glucose is imported by GLUT1 transporter. Glucose is then metabolized by glycolysis into pyruvate. Pyruvate then either enters the TCA cycle through pyruvate dehydrogenase (PDH) for ATP and biomolecule synthesis, or pyruvate is converted to lactate by lactate dehydrogenase (LDH). Lactate is then exported by MCT4 lactate exporter. Extracellular lactate can be imported by MCT1 and converted to pyruvate by lactate dehydrogenase for utilization in the TCA cycle for ATP and biomolecule synthesis. Glutamine is imported by ASCT2 where it then enters the mitochondria and is converted to glutamate. Glutamate can either enter the TCA cycle or is converted to GSH for ROS neutralization. Under metabolic stress conditions, the LKB1-AMPK pathway is activated. LKB1 activates AMPK which inhibits ACC and mTORC1. AMPK inhibition of ACC prevents lipid synthesis and mTORC1 inhibition by AMPK reduces glycolysis. Low glycolysis rate reduces TCA cycle generation of citrate which is used to generate acetyl-CoA for lipid synthesis.

The importance of glycolysis is not only through energy production, but glycolytic intermediates are important for many anabolic processes such as nucleotide synthesis and generating ROS scavengers (Lin et al., 2015; Ju et al., 2017). Cancer cells characteristically show increased utilization of glucose by glycolysis and a shift from OXPHOS to glycolysis to support their growth and division (Warburg et al., 1927). This idea has recently been questioned as OXPHOS was shown to still be utilized for energy production in some cancers (Davidson et al., 2016). OXPHOS is supported by lactate import and utilization through the TCA cycle, while glucose is used to support glycolysis and the pentose phosphate pathway (PPP) shunt to produce ROS scavenger NADPH. NADPH manages ROS stress and is important in generating ribose-5-phosphate for nucleotide biosynthesis (Lin et al., 2015; Ju et al., 2017) (Figures 2,3).

## *LKB1* Regulates Glycolysis

Elevated glycolysis rate is a hallmark of cancer, and tumors deficient in *LKB1* show elevated glycolysis rates, elevated glucose import, and increased expression of glycolytic enzymes. The effect *LKB1* loss has on glycolysis and glucose uptake is mediated by mTORC1 and concomitant oncogenic mutations. In *LKB1* mutant tumors, AMPK activity is diminished which leads to elevated mTORC1 activation (van Veelen et al., 2011). mTORC1 promotes the upregulation of the transcription factor HIF1α that becomes active under hypoxic conditions to promote aerobic respiration and ATP synthesis (Keith et al., 2012) (Figure 2).

HIF1α is regulated both at the protein level and translational level. Under normoxic physiological conditions, HIF1α is targeted for degradation by the E3 ligase von Hipple-Lindau tumor suppressor (Tanimoto et al., 2000). Under hypoxic conditions, HIF1α is stabilized where it translocates to the nucleus and induces gene transcription. Furthermore, mTORC1 promotes the upregulation of HIF1α. This was demonstrated in mouse embryonic fibroblasts (MEFs) by deleting *Tsc2* to promote mTORC1 activity. *Tsc2* ^*−/−*^ MEFs show elevated Hif1α levels, and this was dependent on mTORC1 targets 4Ebp1 and S6k1 (Düvel et al., 2010). In breast cancer cells, mTORC1 promotes HIF1α translation (Sakamoto et al., 2014). The increased mTORC1 activity also correlated with increased glucose uptake and utilization, which was suppressed by depleting *HIF1α*.

*LKB1* mutant tumors show elevated *HIF1α* expression leading to increased glycolysis and lactate production (Andrade-Vieira et al., 2013; Faubert et al., 2014; Nam et al., 2016). Knockdown of *HIF1α* in *LKB1* mutant cells causes reduced growth and cell death under nutrient stress (Faubert et al., 2014; Nam et al., 2016). Gastrointestinal hamartomatous polyps from *Lkb1* heterozygous mice also show increased expression of the glucose transporter *Glut1*, and the first enzyme in glycolysis *hexokinase-2 (HK2)* (Shackelford et al., 2009). Both *GLUT1* and *HK2* are transcriptional targets of HIF1α (Figure 2).

Oncogenic mutations also synergize with *LKB1* loss to promote glycolysis and glucose uptake. *LKB1* is frequently concomitant with *KRAS* in lung cancers and *KRAS* oncogenic mutants upregulate glycolysis. KRAS was shown to promote *HIF1α* expression in colon cancer cells. Furthermore, Kerr et al. showed that *KRAS* copy number impacts glucose utilization (Kerr and Martins, 2018). Increasing the copy number of *KRAS* leads to increased glucose utilization, increased expression of *GLUT* transporters and glycolytic enzymes feeding the TCA cycle, and in later stages, glycolysis mediates management of ROS. Davidson et al. showed that in lung cancer models of *Kras* ^*G12D*^ and *Tp53* (KP mice), *Kras* ^*G12D*^ promotes increased glucose metabolism to generate pyruvate and lactate, consistent with observations that *Kras* oncogenic mutations yield elevated lactate (Davidson et al., 2016). Pyruvate is then shunted to the TCA cycle through pyruvate dehydrogenase (PDH), indicated by increased citrate levels. This indicates that in *Kras* models of lung cancer, glucose is utilized to generate both pyruvate and lactate, and pyruvate is shunted to the TCA cycle. *LKB1* loss can synergize with KRAS oncogenic mutations by increasing glucose uptake and glycolysis, leading to increased lactate production.

## Lactate as an Energy Source in Lung Tumors

Lactate has become more important in energy metabolism than once thought (Hui et al., 2017). Under normal metabolism where OXPHOS is functional, pyruvate is oxidized to acetyl-CoA by the pyruvate dehydrogenase complex (PDC). In hypoxic conditions, pyruvate is converted to lactate by LDH. Cancer cells display elevated *LDH* expression, leading to elevated lactate formation and export. This creates an acidic tumor microenvironment which creates a favorable environment for invasion and metastasis and modulates immune cell functions (Rizwan et al., 2013; Xie et al., 2014; Brand et al., 2016). Furthermore, circulating lactate can be imported and utilized as an energy source and substrate for lipogenesis (Brooks, 2009; Chen et al., 2016). Lactate is imported using the lactate transporter monocarboxylate transporter 1 (MCT1). Inhibiting MCT1 reduces oxidative respiration and promotes an increase in glycolysis in both cancer cell lines in culture and xenograft models (Sonveaux et al., 2008; Pavlides et al., 2009) (Figure 3).

Lactate incorporation in tumor metabolism was seen in human NSCLC patients. NSCLC patients were infused with labeled glucose and incorporation of metabolic intermediates was measured from tumor samples. Results showed elevated lactate labeling compared to glycolytic metabolites (Faubert et al., 2017). Furthermore, *LKB1* mutant xenografts (H460 and HCC15 cells) in mice showed a similar phenotype with increased labeling of lactate (Faubert et al., 2017). In these xenograft models, infusion with labeled lactate showed labeled TCA intermediates, suggesting carbon from lactate can be incorporated into the TCA cycle (Faubert et al., 2017).

This study showed that in H460 and HCC15 cells, lactate shuttled in and out of the cells through lactate transporters. *MCT1* knockout HCC15 cells did not affect viability or division but displayed an increase in oxygen consumption rate (OCR) and decreased ECAR, indicating decreased glycolysis and decreased lactate production and export. This was also seen in HCC15 *MCT1* knockout xenograft models that when exposed to labeled lactate, showed reduced metabolite labeling of pyruvate and TCA intermediates. Like observations in cultured cells, *MCT1* knockout HC115 cell xenografts also displayed reduced glycolysis (Faubert et al., 2017).

This study illustrated the importance of lactate in *LKB1* mutant NSCLC tumors. Lactate can be utilized as an energy source through incorporation into the TCA cycle for ATP generation and TCA intermediates can act as precursors for synthesis of various amino acids. Extracellular lactate from neighboring cells can be imported and incorporated into the TCA through pyruvate (Figure 3). *LKB1* mutant lung cancer cells show elevated LDHA/B and the lactate transporter MCT1/4. The lactate export and import mechanisms highlight the uncoupling of glycolysis from the TCA cycle. Lactate cannot simply be incorporated into the TCA cycle, but rather extracellular lactate is imported for entry into the TCA cycle (Wu et al., 2016; Faubert et al., 2017).

## *LKB1* Regulates ROS Balance: PPP and Glutamine Metabolism

The metabolic reprogramming caused by loss of LKB1 activity results in elevated ROS and metabolic stress. Cellular metabolism generates reactive oxygen species that need to be quenched to prevent damage to DNA, proteins, and RNAs (Finkel, 2011). The increased aerobic glycolysis in cancer cells shunts metabolites towards the PPP to generate nucleotides *via* Ru-5-P and NADPH for ROS scavenging and lipid synthesis (Patra and Hay, 2014). NADPH is also an important mediator of glutathione ROS scavenging. Glutathione (GSH) and thioredoxins (TRX), two proteins that neutralize ROS, are synthesized using NADPH-dependent mechanisms (Nathan and Ding, 2010) (Figure 3). Depleting PPP genes causes increased ROS through defective ROS scavenging and leads to cancer cell death in colorectal cells (Ju et al., 2017). In lung cancer cell lines, depleting 6-phosphogluconate dehydrogenase (6PGD), decreases lipogenesis, RNA biosynthesis and increases ROS (Lin et al., 2015) (Figure 2). Furthermore, in A549 and H460 cells, both *LKB1* mutant cell lines, PPP-related genes are elevated indicating a reliance on the PPP pathway (Martín-Bernabé et al., 2014).

Glutamine is a common metabolite used in ROS scavenging and biosynthesis of biomolecules. Glutamine is essential for cancer cell growth in culture and is the most utilized amino acid. Glutamine can be shunted to the TCA cycle to provide acetyl-CoA or for the synthesis of other biomolecules. This is important in conditions where glucose metabolism generates lactate and not acetyl-CoA like in *LKB1* deficient tumors (Wise et al., 2011; Mullen et al., 2012). *KRAS* oncogene mutations synergize with *LKB1* loss to promote glutamine metabolism to combat ROS (Trachootham et al., 2006; Galan-Cobo et al., 2019). Glutamine can be used for amino acid and nucleic acid synthesis, further pointing to the essentiality for growth in cell culture (Caiola et al., 2016; Gwinn et al., 2018). In lung tumors *in vivo* however, glutamine does not enter the TCA cycle. GLS1, which is required for metabolizing glutamine in mitochondria, is not required for oncogenic *KRAS*-dependent growth (Davidson et al., 2016). As mentioned above, lactate is the primary carbon source for the TCA cycle (Faubert et al., 2017) (Figure 3).

Glutamine in *KRAS* oncogenic mutant tumors is necessary for mediating ROS neutralization. KL tumors are frequently concomitant for *KEAP2*, the inhibitor of oxidative stress response transcription factor NRF2. When *KEAP2* mutations are concomitant with KL mutations, accelerated tumor growth is observed (Romero et al., 2017; Galan-Cobo et al., 2019). Furthermore, when *LKB1* is expressed in *LKB1* deficient A549 cells, cell death is observed due to increased glutamine transformation and are less sensitive than control A549 cells to glutamine inhibitors (Galan-Cobo et al., 2019). *LKB1* mutant cells promote NRF2 dependent *GCL* expression, the primary enzyme to generate *γ*-Gly-Gly from glutamine and cysteine to increase GSH pools. Knockdown of *NRF2* in A549 cells causes decreased GSH formation from glutamine (Mitsuishi et al., 2012). The dependence of *LKB1* deficient cells to glutamine is also seen in polycystic kidney disease where *LKB1* mutant kidneys show increased dependence on glutamine to provide a synthesis of non-essential amino acids and GSH for ROS neutralization (Flowers et al., 2018).

## Hexosamine Biosynthesis

Another pathway that is elevated in *LKB1* mutant tumors is the hexosamine biosynthesis pathway (HBP). The HBP produces UDP-N-acetylglucosamine (UDP-GlcNAc) which is important for protein glycosylation. The HBP integrates intermediates from glycolysis, lipid synthesis, glutamine metabolism, and nucleotide metabolism to generate UDP-GlcNAc (Vasconcelos-dos-Santos et al., 2015; Ferrer et al., 2016). UDP-GlcNAc is used by glycosyltransferases to generate glycoconjugates glycoproteins, glycolipids, and glycosaminoglycans. Glycosylation can modulate protein activity. For example, glycosylation inhibits phosphofructokinase 1 (PFK1) under hypoxic conditions, diverting glycolysis intermediates to PPP to decrease ROS (Sola-Penna et al., 2010).

Lung cancer cells have altered glycosylation and KL tumors display an elevated HBP gene expression profile. *LKB1* acts to suppress HBP, acting as an inhibitor of glycosylation. Inhibiting glycosylation by inhibiting glutamine-fructose-6-phosphate transaminase 2 (GFPT2) causes cell death in KL tumor cells but not in *KRAS* ^*G12D*^ tumor cells. This suggests a potential vulnerability in KL cells that can be exploited (Kim et al., 2020). KL mice treated with GFPT inhibitor Azaserine significantly suppressed tumor growth and this effect was specific to *LKB1* loss. This suggests a potential therapeutic target in KL lung cancers.

## Targeting *LKB1* Mutant Cancers Through Metabolism

The metabolic changes that occur under *LKB1* loss present an opportunity for therapeutic intervention by targeting aberrant metabolic pathways. *LKB1* mutant tumors display elevated glycolysis, lactate metabolism, and fatty acid synthesis due to the loss of AMPK activity, and subsequent loss of mTORC1 inhibition (Carling et al., 1987; van Veelen et al., 2011; Keith et al., 2012; Bhatt et al., 2019). Investigating downstream pathways could identify potential targets for therapeutic intervention. This is the case for biguanides metformin and closely related phenformin. Metformin is used to treat type II diabetes and functions by inhibiting mitochondrial complex I of the ETC (El-Mir et al., 2000; Dykens et al., 2008). The inhibition of complex I uncouples mitochondrial membrane potential, and reduces ATP generation through OXPHOS, leading to activation of AMPK in an LKB1 dependent mechanism (El-Mir et al., 2000; Dykens et al., 2008). In a mouse model of NSCLC where *Lkb1* is deficient, phenformin was able to reduce tumor growth (Shackelford et al., 2013). Although phenformin did not cause activation of AMPK, due to the lack of functional *Lkb1*, the disruption of OXPHOS and subsequent reduction in ATP caused metabolic stress and elevated ROS. *Lkb1* deficient tumors are unable to tolerate high levels of ROS and therefore undergo apoptosis as a result (Shackelford et al., 2013).

Cancer cells rely on elevated glycolysis and overexpress genes related to glucose metabolism (Hanahan and Weinberg, 2011; Pavlides et al., 2009). *LKB1* deficient tumors display elevated glycolysis signature mediated by elevated mTORC1 activity and *HIF1α* expression (Figure 2) (Andrade-Vieira et al., 2013; Faubert et al., 2014; Nam et al., 2016). Inhibitors against mTORC1 have limited therapeutic response in NSCLC. In NSCLC mouse models, the mTORC1 inhibitor rapamycin failed to induce a therapeutic response against KL tumors (Liang et al., 2010). mTORC1 inhibitors are often associated with resistance due to AKT-mTORC2 feedback loop, activating of mTORC1 (Wander et al., 2011). To circumvent potential resistance mechanisms associated with mTOR inhibitors, the Marignani’ group conducted a pre-clinical trial that investigated strategies to inhibit energy and growth simultaneously by targeting glycolysis and mTOR as a potential therapeutic strategy for HER2 positive breast cancer. In *Stk11* ^*−/−*^ */NIC* mice, *Lkb1* loss synergizes with enhanced Her2 activation to promote activation of both arms of mTOR, mTORC1 and mTORC2, resulting in enhanced cell growth fueled by enhanced metabolism. They showed that simultaneously targeting glycolysis and mTOR complexes directly with glucose analog 2-deoxyglucose (2-DG) and mTOR inhibitor AZD8055 reduced tumor growth significantly and provided a stronger effect in combination than either 2-DG or AZD8055 alone (Andrade-Vieira et al., 2014).

Targeting glycolytic enzymes could provide a therapeutic window without dramatically affecting normal cells, which do not rely on elevated glucose metabolism (Pavlides et al., 2009; Hanahan and Weinberg, 2011). WZB117 is a glucose transporter inhibitor that showed promising results in NSCLC xenograft models using *LKB1* deficient A549 cells (Liu et al., 2012). WZB117 reduced ATP, GLUT1 and glycolytic enzyme protein levels (Liu et al., 2012). Furthermore, WZB117 significantly reduced tumor volume (Liu et al., 2012).

The hexokinase-2 inhibitor 2-DG has shown promising results in combination therapies (Maher et al., 2004; Andrade-Vieira et al., 2014). Hexokinase-2 catalyzes the phosphorylation of glucose, restricting its export and keeping glucose inside the cell (Bustamante and Pedersen, 1977; Bustamante et al., 1981). Paclitaxel and 2-DG significantly reduced tumor growth in NSCLC tumor xenograft model compared to single therapy alone (Maschek et al., 2004). By restricting the use of glucose by inhibiting hexokinase-2, other downstream metabolites are also reduced. Glycolysis intermediates cannot be shunted to the PPP to generate NADPH and Ru-5-BP for nucleotide synthesis restricting ROS scavenging and cell division (Figures 2,3). This will increase ROS stress, reduce tumor growth, and induce apoptosis.

*LKB1* mutant tumors are susceptible to ROS stress and rely on multiple pathways to manage ROS stress. *LKB1* mutant tumors display elevated PPP pathway activity to generate NADPH along with increased glutamine metabolism to generate glutathione to scavenge ROS. Treating *LKB1* mutant tumors with compounds that increasing ROS or targeting KL tumors with glutamine inhibitors to reduce glutathione production inhibited tumor growth (Galan-Cobo et al., 2019). Glutamine metabolism is upregulated in KL tumors, where glutamine functions to generate glutathione to manage ROS stress (Figure 3) (Caiola et al., 2016; Davidson et al., 2016; Gwinn et al., 2018). Since *LKB1* deficient tumors are sensitive to increased ROS stress, compounds that produce excess ROS stress could serve as potential therapeutic agents for *LKB1* deficient tumors.

Another potential liability in *LKB1* mutant tumors is lactate metabolism. Similar to the findings by the Marignani group, inhibition of glycolysis can also lead to reduced lactate production. Inhibitors of glycolytic enzymes or LDH can provide potential therapeutic avenues to explore. A549 cells deficient in *LDHA* showed reduced tumor formation after tail-vein injection (Xie et al., 2014). Inhibiting lactate production in KL tumors can restrain oxidative phosphorylation and amino acid synthesis by preventing TCA cycle regeneration. Furthermore, inhibiting lactate transporters could also be an effective approach. Knockout of *MCT1* in *LKB1* deficient cells reduced tumor growth in xenograft models, highlighting lactate metabolism as a potential therapeutic vulnerability (Faubert et al., 2017).

*LKB1* mutant lung tumors display increased fatty acid deposits attributed to the elevated fatty acid synthesis pathways. Loss of AMPK activity causes ACC to become activated and promote fatty acid synthesis, while also impacting the expression of genes involved in fatty acid oxidation (Figures 1,3) (Carling et al., 1987; Bhatt et al., 2019). Targeting fatty acid synthesis by inhibiting ACC in mouse xenografts of *LKB1* deficient cells showed reduced tumor growth (Svensson et al., 2016). Lipids are important components in signaling molecules and for generation of membranes, and fatty acid synthesis is critical for cancer cell growth and survival. Targeting other genes involved in fatty acid synthesis provides other potential targets that could attenuate *LKB1* deficient tumor growth.

Exploiting metabolic vulnerabilities in *LKB1* deficient lung cancer provides promising avenues for the development of novel, effective therapies. Many metabolic pathways are regulated by LKB1. Understanding the impact of *LKB1* loss has on tumor metabolism provides a road map for identifying potential targets.

## Conclusion

Metabolic adaptations by cancer cells are critical to meet the energetic needs and synthesis of macromolecules. Many cancer types show elevated glycolysis even in aerobic conditions. Glycolysis provides intermediates to support nucleotide synthesis and redox balance, both critical to supporting growth and division.

The tumor suppressor LKB1 plays an important role in regulating multiple metabolic pathways. By activating AMPK, LKB1 functions as a metabolic nexus, linking AMPK and mTORC1 signaling, to downstream metabolic pathways. *LKB1* loss is associated with multiple malignancies and causes metabolic reprograming to increase glycolysis and lactate production, elevate hexosamine biosynthesis and glutamine metabolism. Understanding metabolic pathways regulated by LKB1 can highlight therapeutic avenues by targeting pathways dysregulated due to *LKB1* loss and lead to better health outcomes in patients.
